# Antidiabetic Activities of Aqueous Stem Bark Extract of Strychnoshenningsii Gilg in Streptozotocin-nicotinamide Type 2 Diabetic Rats

**Published:** 2012

**Authors:** Sunday Oyedemi, Graeme Bradley, Anthony Afolayan

**Affiliations:** *School of Biological Sciences, University of Fort Hare, Alice 5700, South Africa.*

**Keywords:** Blood glucose, Glucose tolerance, Phytochemical screening, Serum lipid, *Strychnos henningsi*

## Abstract

*Strychnos henningsii *Gilg is recommended among other remedies for the treatment of diabetes in traditional medicine of Southern Africa. The antidiabetic effect of oral administration of aqueous bark extract of the plant at 125, 250 and 500 mg/Kg body weight was investigated in diabetic rats induced with streptozotocin-nicotinamide for 15 days. The extract decreased the blood glucose level, feed and water intake as well as triacylglycerol at the three doses investigated while the best result was obtained at 250 mg/Kg. Similarly, the extract was able to lower the cholesterol level appreciably at 500 mg/Kg while the remaining doses did not have any significant effect as compared with diabetic untreated groups. In addition, the weight loss of diabetic-treated rats was markedly normalized at all doses. The glucose tolerance level of diabetic animals was effectively reduced to near normal level after 90 min of extract administration especially at the dose of 250 and 500 mg/Kg. The phytochemical screening of *S. henningsii *revealed the presence of flavonoids, tannins and saponins which have been reported to increase the insulin secretion. The results obtained from this study demonstrated that the aqueous extract of *S. henningsii *possess antihyperglycemic and antilipidemic properties and thus could prevent various complications of diabetes. Generally, this study has validated the traditional use of this plant for the treatment of diabetes mellitus.

## Introduction

Diabetes mellitus is a group of metabolic disorders resulting from defects in insulin secretion or reduced sensitivity of the tissues to insulin action or both (1). It is characterized through the chronic high blood glucose which causes the glycation of body protein and thus could lead to severe complications (2). Some of these complications are polyuria, polyphagia, polydypsia, ketosis, retinopathy as well as cardiovascular disorders (3). The number of people suffering from this disease is increasing worldwide at an alarming rate. It is projected that 366 million people will be affected by 2030 (4). According to Amos *et al*.(5) about 85-95% of diabetic patients are suffering from type 2 diabetes which is also known as non-insulin-dependent diabetes mellitus (NIDDM). This form of diabetes is very common in people over 40 years of age and the cause has been linked to the high consumption of calorie-rich diet, obesity and sedentary life style (6). It is characterized with the predominant insulin resistance through a relative insulin deficiency or reduced sensitivity of target tissues to the metabolic effects of insulin (7). Unfortunately, the management of this disease is still a challenge to the medical system.

Several drugs such as biguanides, sulphonylureas and insulin have been employed for the treatment of diabetes; however none has been able to cure the disease (8, 9). Moreover, undesirable effects such as hypoglycemic, anorexia nervosa, brain atrophy and fatty liver appear during the intake of oral hypoglycemic synthetic drugs (10). Worse still, the cost of these drugs is beyond the reach of people in the low income group and especially those living in the rural areas. Therefore, there is a need to search for new and affordable remedies for diabetes. Recently, the search for antidiabetic agents has been focused on plants because of their ready availability, affordability, effectiveness and probably due to their low side effects. However, few of these plants have received scientific or medical scrutiny and World Health Organization has recommended further evaluation of traditional plants used for the treatment of diabetes (11). One of the plants widely used in traditional medicine for the treatment of diabetes mellitus in southern Africa is *Strychnos henningsii*.

*S*. *henningsii *Gilg. (Loganiaceae) is a small evergreen tree or shrub with leathery leaves. The bark is crown compact with dark green, glossy foliage and the fruit is oblong which turns brown when ripen. It is one of the most widely distributed species of strychnos in east and southern Africa (12). The leaves of the plant have a characteristic aromatic-pungent odor with a rough texture. The decoctions or infusions of this plant have been recommended by traditional health practitioners in southern Africa for the treatment of various diseases. These include rheumatism, gynaecological complaints, abdominal pain, snake bite, gastrointestinal pain, malaria and diabetes mellitus (13, 14). Phytochemical screenings conducted in this study have shown that *S. henningsii *contains tannins, saponins, alkaloids, flavonoids, steroids, triterpenes and cardiac glycoside. About five compounds, including retuline-like alkaloids, strychnine, brucine, curarine and bitter glycoside have been isolated from this plant (15).

Before the commencement of this work, there was no information in scientific literature about the antidiabetic effect of aqueous bark extract of *Strychnos henningsii *in streptozotocin-nicotinamide induced type 2 diabetic rats. Therefore, the objective of this study was to investigate the scientific basis for the use of this plant in the management of diabetes mellitus using the animal model.

## Experimental

*Plant material*

The bark of *S. henningsii *was collected in February, 2009 from a thick forest in Amathole District (Eastern Cape, South Africa). The plant was identified through its vernacular name and later authenticated by Prof. D.S. Grierson of Botany Department, University of Fort Hare. Voucher specimen (Sun MED 2009) was deposited at the Giffen Herbarium of the University.

*Preparation of the extract*

The bark material of *S. henningsii *was air-dried at room temperature in the laboratory. The dried material was then pulverized using an electric blender (Waring Products Division, Torrington, USA). About 60 g of the powdered material was extracted in 1 L of cold distilled water maintained on a mechanical shaker (Stuart Scientific Orbital Shaker, UK) for 48 h. The extract was filtered using a Buchner funnel and Whatman No.1 filter paper. The filtrate was quickly frozen at -40°C and dried for 48 h using a freeze dryer (Savant Refrigerated vapor Trap, RV T41404, USA) to give a yield of 8.4 g of dry extract. The resulting extract was reconstituted in distilled water to give desired doses (125, 250 and 500 mg/mL) used in this study.

*Phytochemical screening*

The aqueous bark extract of *S. henningsii *was subjected to phytochemical analysis to determine the presence of bioactive compounds such as phenols, flavonoids, alkaloids, saponins, glycosides and tannins using the general chemical test of Zafar and Mujeeb (16).

*Assay kits and reagents*

The assay kits for the analyses of triacylglycerol and cholesterol were obtained from Randox Laboratories Limited, Ardmore, Co Antrim, UK. All other reagents used were of analytical grade and were supplied by Merck Chemicals (Pty) Ltd. (Merck, Bellville, South Africa).

*The animals*

Male Wistar rats (*Rattus norvegicus*) weighing 125-255 g were obtained from the animal house of the Agricultural and Rural Development Research Institute, University of Fort Hare. They were kept in well-ventilated house conditions (temperature: 28 ± 1°C; photoperiod: 12 h light/12 h dark cycle; humidity: 45-50%). The animals had free access to food and water *ad libitum *for 15 days. The experiment was approved by the Animal Ethics Committee of the University of Fort Hare.

*Induction of type 2 diabetes in rats*

The method of Pellegrino *et al. *(17) was adopted for the induction of type 2 diabetes mellitus in overnight fasted Wistar rats. The animals were induced by a single intraperitoneal injection (IP) of freshly prepared solution of streptozotocin (60 mg/Kg body weight) in 0.1 M citrate buffer (pH 4.5), 15 min after the intraperitoneal administration of nicotinamide (110 mg/Kg) prepared in normal saline. Diabetes was confirmed in the animals by the elevated plasma glucose levels after 24 h of injection. The rats with diabetes having glycosuria and hyperglycemia (blood glucose > 8.1 mmol/L) were used for the experiment.

*Animal grouping and extract administration*

Thirty six male rats were randomized into six groups of five animals each (30 diabetic surviving rats, 6 normal rats). Group I: normal control rats orally administered with daily drinking water for 15 days using gavage; group II: diabetic animals received 0.5 mL of distilled water; group III-V: diabetic rats treated daily with 0.5 mL of 125, 250 and 500 mg/Kg body weight of *S. henningsii *extract respectively; group VI: diabetic animals received 0.5 mL of glibenclamide only. All the animals from each group were sacrificed by ether anesthesia 24 h after their respective 15 daily doses of the extract and distilled water.

*Oral glucose tolerance test (OGTT)*

Thirty six rats (normal) were fasted for 12 h and assigned randomly into 6 equal groups (n = 6/group). They were fed orally with aqueous bark extract of *S. henningsii *at the doses of 125, 250, 500 mg/Kg and glibenclamide (0.6 mg/Kg) using gavages. The remaining groups consisting of diabetic and normal rats were treated orally with distilled water. Glucose (2 g/Kg) was orally administered 30 min prior to the extract administration and blood was withdrawn from the tail vein at 30, 60 and 90 min (17). The fasting plasma glucose level was measured using glucometer (Bayer Health Care, Japan).

*Serum lipid profile*

The method described by Tietz *et al. *(18) was adopted to assay the cholesterol and triacylglycerol in the serum of the animals. They were measured spectrophotometrically using assay kits from Randox Laboratories Limited, Ardmore, Co Antrim, UK.

*Effect of extract on the weight, feed and water intake of rats*

Feed and water intakes were measured everyday at the same hour during the experimental periods while the body weight of the animals were taken gravimetrically before the start and every fifth day throughout the experimental period (15 days).

*Statistical analysis*

Data were expressed as means of six replicates ± SD and were statistically analyzed using one-way analysis of variance (ANOVA). Means were separated by the Duncan multiple test using SAS. Values were considered significant at p < 0.05.to paired Student’s t-test. Significant levels were tested at p < 0.05.

## Results and Discussion

The qualitative phytochemical analysis of the aqueous stem bark extract of *S. henningsii *revealed the presence of tannins, flavonoids, terpenes, saponins, steroids and alkaloids while cardenolides and dinolides were absent (Table 1). These compounds have been reported to elicit a wide range of biological activities such as insulin-like effect, anti-hypercholesterol and hypotensive activity (20-21). For example, saponins are well-known to lower serum cholesterol by converting it to bile acids. The hypoglycemic and hypolipidemic properties of alkaloids, flavonoids and tannins have also been reported (22). The presence of these compounds might contribute to the antidiabetic effect of this plant as observed in the present study.

**Table 1:** Phytochemical analysis of aqueous extract of *S. henningsii *bark

**Phytochemical compounds**	**Plant extract**
**Alkaloids**	+
**Tannins**	+
**Saponin**	+
**Flavonoids**	+
**Cardiac glycosides**	+
**Antraquinone**	+
**Phenolics**	+
**Steroids**	+
**Triterpenes**	+
**Cardenolides and Dinolides**	-

**+**: Presence; **-**: Absence

The glucose tolerance test was carried out to verify the glycemic control of the plant’s extracts after the treatment. The results of glucose tolerance test in diabetic, control and normal rats treated with *S. henningsii *and glibenclamide after oral administration of glucose for 30, 60 and 90 min were shown in Table 2.

**Table 2:** Effect of oral administration of aqueous extract of *S. henningsii *on oral glucose test in male Wistar rats

**Treatments**	**Oral glucose tolerance (OGTT)**			
	**0**	**30**	**60**	**90**
**Normal control**	4.60 ± 0.40	4.90 ± 0.53	4.40 ± 0.30	4.13 ± 0.32
**Diabetic control**	14.73 ± 8.00^a^	18.63 ± 7.00^a^	21.64 ± 7.20^a^	20.04 ± 5.10^a^
**Normal + SH (125 mg/Kg)**	5.50 ± 1.60^b^	10.40 ± 1.59^b^	18.60 ± 2.42^b^	15.77 ± 2.84^b^
**Normal + SH (250 mg/Kg)**	5.14 ± 2.20^b^	6.87 ± 2.30^c^	10.20 ± 2.40^c^	7.93 ± 1.93^c^
**Normal + SH (500 mg/Kg)**	5.47 ± 2.00^b^	7.10 ± 0.92^d^	7.50 ± 1.98^d^	7.53 ± 1.98^c^
**Normal + glibenclamide (0.6 mg/Kg)**	4.40 ± 2.50^c^	5.03 ± 1.59^d^	4.07 ± 2.42^e^	4.60 ± 2.84^d^

Values are mean ± SD of 6 rats in each group. ^a-e^Test values carrying superscripts different from the control for each parameter are significantly different (p < 0.05). Difference in blood glucose of each group (final-initial values): Diabetic (↑5.31 mmol/L); 125 mg/Kg (↓2.83); 250 mg/Kg (↓2.97 mmol/L); 500 mg/Kg (↑0.03 mmol/L); Glibenclamide suppressed increased blood glucose near normal. Where ↑ means increase and ↓ represents decrease.

 At 30 min, after the glucose administration, the peak of blood glucose level was increased rapidly from the fasting glucose value and then subsequently decreased. Plant extracts at all the three doses exhibited noticeable blood glucose lowering effect at 90 min. The dose of 250 and 500 mg/Kg body weight showed a similar blood glucose lowering capacity, whereas, that of glibenclamide-treated rats showed a comparable result to that of normal control rats. The significant reduction of the peak levels of blood sugar within 90 min manifests the antidiabetogenic potential of *S. henningsii *extract in rat models. This result showed an appreciable improvement of the glucose tolerance test which could be attributed to the insulin mimetic activity of the plant’s extract by restoring the delayed insulin response (23).

Streptozotocin (STZ) is well known for its selective pancreatic islet beta cell cytotoxicity and has been extensively used to induce type 1 diabetes mellitus in animals (24). However, for type 2 diabetes mellitus to be induced, nicotinamide was injected into the rats intraperitoneally to activate the poly ADP ribose synthase in order to repair the damaged DNA caused by STZ (25, 26). This method has been used to induce diabetes mellitus in animals that resemble non-obese type 2 diabetes mellitus that constitutes a majority of East Asian diabetic patients (27). Prior to the STZ induction into the rats, blood glucose levels did not differ significantly between groups but increased nearly 3.5 fold after 24 h of induction as compared to the normal control rats.

The Streptozotocin Nicotinamide (STZ- NAD) diabetic rats elicited a significant rise in plasma glucose level from 18.2 to 30.1 mmol/L for 15 days of experimental period (Table 3). 

**Table 3:** Effect of oral administration of aqueous extract of *S. henningsii *on plasma glucose level of STZ-induced diabetic rats

**Treatment**	**Plasma blood glucose (mmol/L)**			
	**0 (day)**	**5 (day)**	**10 (day)**	**15 (day)**
**Normal control**	5.60 ± 0.40	5.40 ± 0.53	5.6 ± 0.30	5.70 ± 0.32
**Diabetic control**	18.20 ± 0.30^a^	23.45 ± 0.35^a^	28.30 ± 0.40^a^	30.10 ± 0.40^a^
**Diabetic + SH (125 mg/kg)**	19.33 ± 1.20^a^	16.35 ± 1.14^b^	15.53 ± 1.30^b^	12.56 ± 0.90^b^
**Diabetic + SH (250 mg/kg)**	24.30 ± 0.09^b^	22.20 ± 0.08	17.57 ± 1.02^c^	14.27 ± 1.20^c^
**Diabetic + SH (500 mg/kg)**	25.30 ± 0.01^b^	20.20 ± 0.04^c^	19.28 ± 0.10^d^	17.70 ± 0.30^d^
**Diabetic + glibenclamide (0.6 mg/kg)**	22.50 ± 3.30 ^c^	19.30 ± 3.20^c^	17.78 ± 2.40^c^	13.52 ± 2.50^c^

Values are mean ± SD of 6 rats in each group. ^a-e^ Test values carrying superscripts different from the control for each parameter are significantly different (p < 0.05). Difference in blood glucose of each group (final-initial values): Diabetic (↑11.9 mmol/L); 125mg/Kg (↓6.7); 250 mg/Kg (↓10.03 mmol/L); 500 mg/Kg (↓7.60); Glibenclamide (↓8.98 mmol/L). Where ↑ means increase and ↓ represents decrease.

The observed increase in blood glucose as reported by (28) was attributed to the abnormalities in pancreatic beta cell and thus affects the insulin secretion. Meanwhile, the administration of aqueous bark extract of *S. henningsii *at all the three doses significantly reduced the blood glucose level from 13 to 6 mmol in a dose-independent manner. The difference between the treated groups and diabetic control rats in lowering the fasting plasma glucose levels was significant. The higher dose (500 mg/Kg) did not produce a stronger effect as expected in this study. This was likely due to the presence of other substances that might have interfered with antidiabetic property of this plant. However, the dose at 250 mg/kg (13 mmol) exhibited more potent antihyperglycemic potential than other treated groups throughout the experimental period. This result confirms the ability of the plant to potentiate the insulin secretion from the existing beta cells or by its release from the bound form as shown in glucose tolerance test.

After the streptozotocin injection, the serum cholesterol and triacylglycerol of diabetic rats increased significantly above the normal level. The marked hyperlipidemia that characterizes the diabetic state may therefore be regarded as a consequence of the uninhibited actions of lipolytic hormones on the fat depot (29). This elevation can give useful information on the predisposition of animals to secondary complications of diabetes including atherosclerosis and its associated coronary heart disease (30). However, after the extract administration, the level of serum triacylglycerol was significantly reduced at the investigated doses (Table 4) while that of cholesterol was only reduced at the dose of 500 mg/Kg. Meanwhile, the levels of cholesterol and triacylglycerol in untreated diabetic group remained elevated throughout the experimental period which is in agreement with the finding of Shanmugasundaram (31).

**Table 4:** The effect of aqueous extract of *S. henningsii *on the cholesterol and triglyceride level in STZ-induced diabetic rats

**Serum lipids parameter**	**Control**	**Diabetes control**	**D**_o_	**D**_1_	**D**_2_	**D**_3_
**Cholesterol (mmol/L)**	1 .57 ± 0.12^a^	6.67 ± 0.05^c^	5.15 ± 0.16^b^	6.67 ± 0.05^c^	6.38 ± 0.00^c^	4.60 ± 0.12^d^
**Triacylglycerol (mmol/L)**	0.78 ± 0.34^a^	2.17 ± 0.33^b^	0.58 ± 0.57^c^	0.65 ± 0.30^d^	0.73 ± 0.00^d^	0.50 ± 0.22^e^

Values are mean ± SD of 6 rats in each group. ^a-e^ Test values carrying superscripts different from the control for each parameter are significantly different (p < 0.05). D_o_ = Diabetic + SH (125 mg/Kg), D_1_ = Diabetic + SH (250 mg/Kg), D_2_ = Diabetic + SH (500 mg/Kg) and D_3_ = Diabetic + glibenclamide (0.6 mg/Kg).

 The result obtained from this study showed that the aqueous bark extract of *S. henningsii *possesses triacylglycerol lowering ability. This could be attributed to the presence of antilipidemic compounds that may inhibit or activate some enzymes involved in triacylglycerol metabolism (32).

**Figure 1:**
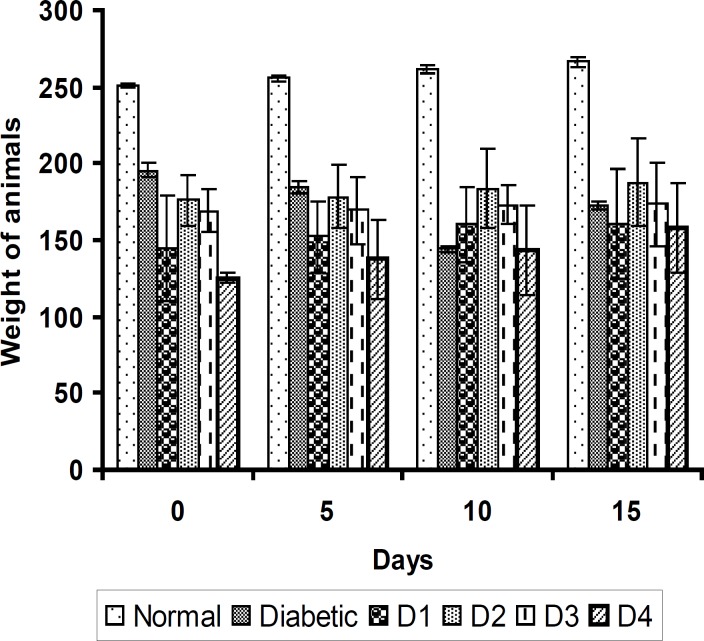
The effect of aqueous extract of *S. henningsii *(SH) on the body weight of diabetic rats. Values are mean ± SD of 6 rats in each group. D1 = Diabetic + SH (125 mg/Kg), D2 = Diabetic + SH (250 mg/Kg), D3 = Diabetic + SH (500 mg/Kg) and D4 = Diabetic + glibenclamide (0.6 mg/Kg).

 The induction of diabetes with STZ-NAD is associated with a characteristic loss of body weight (↓33 g) as shown in Figure 1.

**Figure 2:**
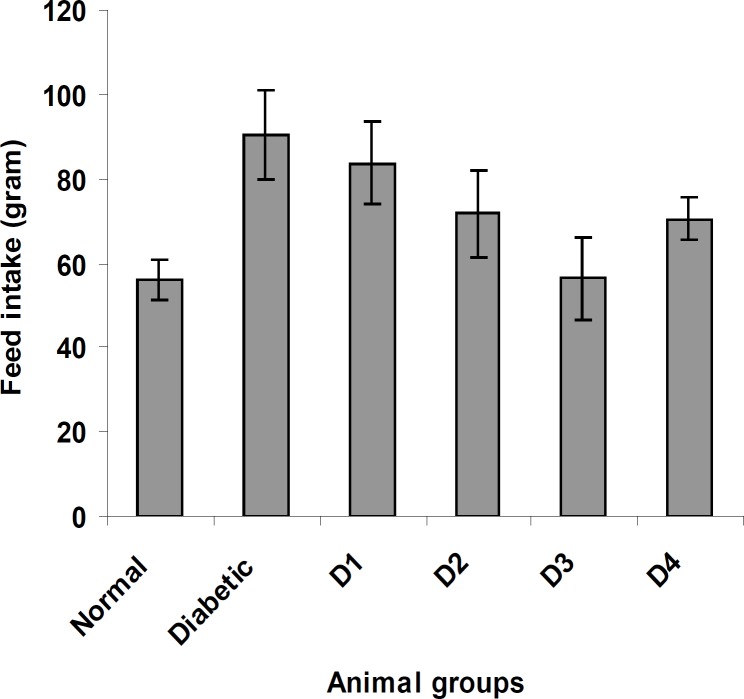
The effect of aqueous extract of *S. henningsii *on the feed intake of diabetic rats. D_o_ = Diabetic + SH (125 mg/Kg), D_1_ = Diabetic + SH (250 mg/Kg), D_2_ = Diabetic + SH (500 mg/Kg) and D_3_ = Diabetic + glibenclamide (0.6 mg/Kg).

**Figure 3:**
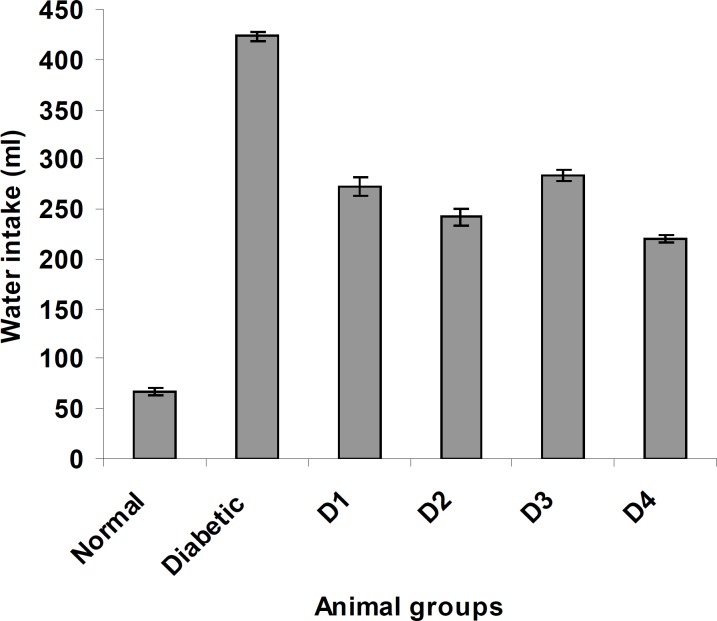
The effect of aqueous extract of *S. henningsii *on the water intake of diabetic rats. D_o_ = Diabetic + SH (125 mg/Kg), D_1_ = Diabetic + SH (250 mg/Kg), D_2_ = Diabetic + SH (500 mg/Kg) and D_3_ = Diabetic + glibenclamide (0.6 mg/Kg).

 Similarly, the feed and water intake of the diabetic rats were significantly increased in comparison with the normal control rats (Figures 2 and 3 respectively). These symptoms are well-known markers of type 2 diabetes in both human and animal models which are direct consequence of insulin deficiency (33). The daily administration of the plant extract to diabetic rats for 15 days caused a significant increase and decrease in body weight and in feed and water intake, respectively, which was an indication of proper glucose utilization in the animals. All three doses of the plant extract improved the body weight of the diabetic rats. Meanwhile, the dose at 125 mg/Kg body weight (16.48 g) was significantly higher than other treated groups. The observed results could be attributed to the improved glycemic control and the protective effect of the plant’s extract in controlling the muscle wasting and induced adipogenesis (29).

In conclusion, the present study demonstrated that the administration of the aqueous bark extract of *S. henningsii *possesses antihyperglycemia and antihypertriacylglycerol property and could also ameliorate various complications of diabetes mellitus. This effect may be due to the presence of bioactive compounds that potentiate the secretion of insulin and protect pancreatic *β*-cell from degeneration. Further studies are currently underway to confirm the mechanism of this plant’s action.
